# In Between Digital War and Peace

**DOI:** 10.1080/15027570.2024.2415201

**Published:** 2024-10-22

**Authors:** Jasmijn Boeken

**Affiliations:** a Institute of Security and Global Affairs (ISGA), Leiden University, Den Haag, The Netherlands; b Centre of Expertise Cyber Security, The Hague University of Applied Sciences, Den Haag, The Netherlands

**Keywords:** Cyberwar, Walzer, cyberspace, digital attacks, just war theory

## Abstract

The world can be divided into a digital sphere and a physical sphere. Within the realm of the physical sphere, Michael Walzer’s Just War Theory stands as a prominent framework for understanding the ethics of warfare. Is his theoretical framework also applicable to the digital sphere? This article studies whether elements of Walzer’s theory can be adapted to the context of digital conflict. Walzer divides countries into zones of peace, zones of war, and in-between zones. A country could then, for example, be a physical zone of peace but a digital zone of war. This article explores in which ways the defining characteristics of the different zones can be found in the digital sphere. It concludes that the digital sphere should mostly be seen as an in-between zone, with the problem of very unclear borders. Regarding Walzer’s theory, while it offers valuable insights when applied to the digital sphere, difficulties arise, underscoring the necessity for further research to refine the principles of just war within the digital sphere.

## 1. Introduction

In an age where everything is becoming increasingly digital, so is war. Recently, at the same location where, over a decade ago, the first major cyberattack hit, another attack happened (Corera 2021). A power blackout at a nuclear enrichment plant received global attention. According to Iran, the attack was made by Israel, however, as is typical of cyberattacks, the attribution of the act remains ambiguous. The digital sphere has become a new domain of warfare, supplementing the traditional arenas of land, sea, air and space (Taddeo 2012). This new addition to warfare has prompted scholars to explore the ethical implications of cyberwar, using frameworks such as Just War Theory (Boylan 2013; Sleat 2018; Taddeo 2012).

As is well-known to readers of this journal, Just War Theory is concerned with the question of what is ethical in warfare (Fotion 2007). The traditional paradigm of just war has developed over many centuries and is today known not least for Michael Walzer’s contributions (with Walzer 1977 as the paradigm text; see also Boylan 2013). Applying it to the topic of cyberwar, scholars have found multiple issues to discuss, such as the identification of combatants and non-combatants (Taddeo 2012), the issue of attribution (Boylan 2013), non-human targets (Sleat 2018), and the fact that the “ground” in cyberspace is continuously increasing (Singer and Friedman 2014). The various scholars have also posed their possible solutions, such as changing the architecture of the internet (Boylan 2013), applying information ethics (Taddeo 2012), or adjusting Just War Theory to make it appropriate for the topic of cyberwar (Sleat 2018). There is, however, yet to be a widespread consensus on the definition of cyberwar and the ethical parameters guiding it.

Although Walzer’s Just War Theory has been applied to the context of cyberwar in various scholarly works, a short 2007 article from Walzer remains unexplored. In this article, Walzer distinguishes zones of peace, zones of war, and in-between zones. While zones of peace are characterized by the rules of a constitutional democracy, in zones of war there are soldiers in the streets and physical unrest. Walzer explains how Yemen, at the time, was neither a zone of war nor a zone of peace, but an in-between zone, lacking both a robust constitutional democracy and active military conflict. The rules that apply there were neither those of peace nor those of war, as described in Walzer’s just war framework.

This article explores whether the cyberspace realm should be considered a zone of war, a zone of peace, or an in-between zone.

What is essential here is the distinction between the digital sphere and the physical sphere. Although the United States might be a physical zone of peace, it could possibly be a digital zone of war at the same time. This argument assumes that cyberattacks are mainly digital, with little effects on our physical world (Dipert 2016). The prospects are, however, that in the future, the physical and digital worlds will become increasingly intertwined. This does not eliminate the importance of the discussion in this article but underlines it; if we want to be able to make sense of the complexities of the digital and physical world, we need to gain a better understanding of the digital world itself.

The article discussed here (Walzer 2007), was originally intended to address emergent challenges in modern warfare brought on by a growing amount of terrorist groups and the war on terror. I believe it furnishes a potential contribution for another novel challenge: cyberwar. Furthermore, Marsili (2019) suggests that cyberwar and cyberterrorism may share similarities, with the demarcations between these concepts remaining ambiguous. Additionally, parallels exist between the issues described by Walzer regarding the war on terror and those inherent in cyberwar, particularly regarding the lack of clarity on the rules governing these domains.

My article is divided into four sections. In the first section, the concept of cyberattacks, the different types of cyberattacks, and the analogies we should use when comparing it with physical war are discussed. The second section covers a discussion of whether the rules of a constitutional democracy, which govern peace zones, are present in the digital sphere. In the third section, we ask whether the digital sphere should be seen as a zone of war by looking at digital acts of aggression and the digital equivalent of soldiers on the ground and in the streets. In the final section, it is considered whether the in-between zone exists in the digital sphere and whether this might be a solution to the theoretical problems of the other zones. This article will be concluded by discussing whether and why Just War Theory can face the challenge of cyberwar.

## 2. Cyberattacks: definitions, shapes and sizes

The terms “cyberwar” and “cyberattacks” are widely used but interpreted in a variety of ways across scholarly works. In this article, the term cyberwar is used to indicate that the topic of discussion is warfare in cyberspace, the digital sphere, as opposed to conventional war, which takes place in the physical sphere. Whether or not cyberattacks constitute cyberwar will be further discussed in the third section. Initially, it is important to establish a precise definition of what constitutes a cyberattack. One of the most widely used definitions of a cyberattack is the one used by Dipert (2016), which states that all attacks on the governmental or civilian information systems of another nation can be grouped as cyberwarfare attacks. However, as Beard (2013) rightfully notes, this also includes the bombing of a building where digital infrastructure is hosted. Given the distinction between the digital and physical sphere in this article, Dipert’s definition is therefore deemed incompatible. In contrast, Beard’s definition, which describes a cyberattack as “the use of computer software and technology by one nation to attack the governmental or civilian information systems of another nation” (Beard 2013, 2) aligns more closely with this digital-physical distinction. Thus, while acknowledging the potential for digital actions to affect the physical sphere, this article adopts Beard’s narrower, digitally focused definition.

With a working definition of a cyberattack now established, it is necessary to delineate the various shapes such attacks can take. Dipert (2016) distinguishes three: (1) Denial of Service (DoS) attacks, (2) Distributed Denial of Service (DDoS) attacks, and (3) intrusive attacks. In the case of a DoS attack, the target (be it a site or a server) gets a large number of requests for service every second. The goal of this sort of attack is to make the target inaccessible for the people that want to use it (Dipert 2016). The second type of attack is a DDoS attack, the goal of which is the same as with the DoS attack, but it is more efficient because it launches from different computers. The third kind of attack is an intrusive attack through malware. The goal of this attack can be many things: crash the system, erase information, send emails, and much more. Most of these intrusive attacks do not cause immediate harm but are used to gather data. According to Dipert (2016), this means that these are not cyberweapons in themselves, but the data that is gathered can be used as weapons.

There are thus different types of cyberattacks, and their impact can be of minor or major influence, depending on their impact and intended target. Minor attacks tend to cause temporary disruptions without long-term consequences, whereas major attacks pose severe threats to national security and can have physical, real-world effects (Michael et al. 2010). However, while data theft might be considered of minor influence, continuous data harvesting could have serious consequences. This distinction between minor and major attacks and their role in cyberwar remains ambiguous and should be discussed in future research.

In cyberwar, there are three possible military targets: a cyberattack can target political/military communication, weapon systems, or infrastructure (Dipert 2016). For example, the Stuxnet attack on Iran’s nuclear facilities can be seen as an attack on the country’s weapon systems; it was a cyber missile (Farwell and Rohozinski 2011). However, while cyberattacks obviously can take the form described above, most of them contain the theft of personal data of citizens (Corera 2021; Dipert 2016). When data theft is compared to the effects that a physical attack can have, it might seem insignificant. In her book *The Age of Surveillance Capitalism,* Zuboff (2023), however, argues that data is the new oil. This insight could provide us with an interesting new analogy. Where during a physical war, oil facilities are important strategic sites to bomb or take over, this might, in a digital war, take the form of data theft. While oil is of course economically important, it is particularly important in war because of its connection with weapons; for instance, oil dependency can affect decisions regarding arms trade (Bove, Deiana, and Nisticò 2018). A counterargument to this analogy could be that data is not something that can be turned into a weapon. However, that argument could not be more wrong. For example, biometric data like facial patterns are an important source for developing facial recognition software. As Amoore (2006) describes in an article on terrorism, this data can be used in fighting the war on terror when it comes to, for example, border security. One could also think about the use of facial recognition software for drones, to identify targets (Melkumyan and Mkrtchyan 2023).

This discussion shows the variety in cyberattacks and the similarities and distinctions between physical and digital attacks, leading us towards a nuanced discussion on zones of war, peace and in-between in the subsequent sections.

## 3. Peace in cyberspace

Walzer (2007) argues that countries can be categorized into zones of peace, zones of war, and in-between zones, each with their own ethical rules about which behavior is just and which is not. A zone of peace is where there are no armies on the ground and, ideally, the rules of a constitutional democracy are honored. While most Western countries are physical zones of peace, this section discusses whether we can at the same time consider them digital zones of peace. This comes down to the question of whether the rules of a constitutional democracy are honored in the digital sphere.

According to Walzer (2007), in a peace zone there is, ideally speaking: (1) limited power for the police, (2) respect for democratic freedoms, and (3) no invasion of the privacy of citizens. Before looking at these peace-zone rules in the context of cyberspace, it must be noted that future work could cover a more thorough evaluation of these points, as this article merely provides an exploratory analysis. The powers of the police in constitutional democracies are limited both offline and online, honoring the first rule of a peace zone (Marx 2001). Respect for democratic freedoms is increasingly becoming an important discussion for the digital sphere. A recent example is the European Union Directive on Copyright in the Digital Single Market (the CDSM Directive), which prescribes the use of “upload filters.” The controversial Article 17 of the new EU Copyright Directive requires filters to block all photos, videos, and texts that cause a suspicion of copyright infringement (Heldt 2019). Introducing such filters, however, raises questions about free speech and censorship in the digital sphere (Marx 2001).

The final characteristic of a peace zone that Walzer (2007) emphasizes is the protection of citizens’ privacy. While states do regulate police conduct in the digital sphere, what is of principal concern here is the behavior of companies, as highlighted by Zuboff (2023), who contends that our digital privacy remains dangerously unprotected. Contrarily, Boylan (2013) views privacy as a digital hazard that impedes attribution and suggests that this may lead to a war of all against all in the digital domain. Boylan proposes to redesign the internet with mandatory identification and tracking to keep it secure. However, I concur with Walzer, arguing for the imperative of privacy preservation as a cornerstone of digital peace.

The significance of privacy spans several domains: it is crucial for personal freedom and autonomy, as it ensures control over personal information (Schwartz 1989; Van den Hoven 1997); it underpins political sovereignty by safeguarding citizens against echo chambers that skew our perceptions and decisions (Sunstein 2018); it fosters intimate bonds, with information sharing as a key element of developing and maintaining friendships (Fried 1984; Rachels 1975); and it is essential for our very humanity, as the private life should not involuntarily become public (Arendt 1958; Borren 2010). These perspectives reinforce Walzer’s contention that privacy is indispensable for peace, especially in our data-centric digital sphere. As with our analogy to oil above, the protection of data is of paramount importance.

The question that this article seeks to answer is whether the digital sphere is a zone of peace, a zone of war, or an in-between zone. So far, I have suggested that we cannot simply classify the digital sphere as a peace zone. Let us now consider whether the digital sphere should, by contrast, be considered a zone of war.

## 4. War in cyberspace

In the preceding section, it was established that in the digital sphere, the rules of a peace zone are not obviously applicable. This analysis now turns to the question of whether the digital sphere should be considered a zone of war instead, considering (1) the presence of states’ “political sovereignty” and “territorial integrity” in the digital sphere, and (2) whether digital attacks can be acts of aggression (Walzer 2007). What is important to recognize is the fact that the occurrence of digital acts of aggression does not necessarily render a country a digital zone of war. As Walzer (2007) rightfully notes, when the United States was at war with the Taliban, the United States itself was not a war zone. It is important, in other words, to note the distinction between a *zone* of war and a *state* of war. Whereas the US was thus not a zone of war, it was arguably in a state of war with terrorism or with certain terror groups.

Walzer’s (1977, 2007) description of Just War Theory rests on the idea of a domestic analogy. In short, a sovereign state is formed of a political community, which consists of citizens’ experience and cooperative activity over a long period of time. Furthermore, citizens are granted the individual right to “life” and “liberty,” reflecting what it means to be a human being. According to Walzer, then, the legitimacy of states is based on the consent of their members, and therefore, it is the state’s duty to protect its political community from external encroachment. If a state is being attacked, the citizens’ individual rights to life and liberty are under attack (Walzer 2007). Therefore, states are, just like individuals, granted a set of rights to protect the political community. These are “political sovereignty” and “territorial integrity.” Any violation of a state’s political sovereignty or territorial integrity is by Walzer labeled as an act of “aggression.”

Identifying a violation of a state’s political sovereignty and territorial integrity in the digital sphere is a challenging task. According to Walzer, when a state is sovereign this means that they are free from foreign coercion and control (Walzer 1977). A cyberattack that manipulates election results is an instance of a violation of a state’s digital sovereignty. Take as an example Russia’s interference in the 2016 United States elections (Ziegler 2018). Attacks that target the political sovereignty of a state are specifically aimed at coercing and controlling citizens (Smotherman 2016). In the case of the 2016 US elections, they aim to spread doubt about the legitimacy of democratic institutions (McCombie, Uhlmann, and Morrison 2020).

But how, then, should we classify a cyberattack like data theft, following Walzer’s theory?

Inspired by Walzer, Farwell and Rohozinski (2011, 30) describe a cyberattack as “the use of cyber weapons that cause damage to property or injury to human beings.” This leads to the argument that attacks where data is stolen are not an act of aggression. This is also the essence of Dipert’s (2016) criticism of Just War Theory. However, as Beard (2013) rightfully noticed, authors such as Dipert, Farwell, and Rohozinski do not adequately cover Walzer's conception of aggression. Walzer does not merely see a physical attack as an act of aggression, but as described above: “the breach of territorial integrity and/or political sovereignty” (Beard 2013, 5). Data theft can thus be an invasion of the digital safe space of citizens; hence it violates the territorial integrity of that state. Just War Theory’s view on acts of aggression is, when considered broadly, applicable to cyberwar as well.

However, the question is whether we can divide the digital sphere into zones of peace and zones of war. Walzer (2007) describes a war zone as a place where soldiers walk the streets. This description of a war zone is a valid target for the critique from Dipert (2016); this only considers physical attacks. For the digital sphere, this would mean that only attacks that damage property or injure citizens could make a country into a digital war zone. However, as described in the first section, the effects of data theft are very real, and they must be taken seriously. We must therefore question whether Walzer’s (2007) definition of a war zone as “boots on the ground” is adequate for dividing the digital sphere into zones of peace and war, as it does not consider certain types of cyberattacks that do not directly damage property or physically injure citizens. So, although data theft can be characterized as an act of aggression it does not immediately turn the digital sphere into a zone of war.

While Walzer’s theory can be criticized for being unsuited in properly identifying digital attacks, it becomes evident that when you take a broader view of the theory, it is still applicable to this new form of war. However, the conception of a war zone is very limited. It leads to the conclusion that we cannot consider most Western countries to be a digital zone of war. However, as the previous section showed, they are not a zone of peace. I believe we must delineate it as an “in-between zone.”

## 5. In-between digital war and peace

In a report from the Dutch Security and Justice Ministry (Ministerie van Justitie en Veiligheid, 2020), the digital sphere is described as a gray area between peace and war. This is what Walzer (2007) would describe as an in-between zone.

As Walzer (2007) describes it, there are no clear-cut rules in the in-between zones about how to respond to acts of aggression. In his article, Walzer uses the example of Yemen. In Yemen at that time, there were no armies constantly fighting each other, and no soldiers in the streets; hence it could not be considered a zone of war. But it was also not a zone of peace, as the “state’s writ does not run in the desert of South Yemen” (Walzer 2007, 481). There were no policemen on the streets who kept the country stable, and the Yemeni government was not acting effectively. The US-targeted killing of Al-Qaeda soldiers in South Yemen, therefore, brings a difficulty to light. The US was not at war with Yemen and could therefore not claim combatant rights according to international law and just war theory. At the same time, Al-Qaeda terrorists did pose a general threat and hence there was a responsibility to act (Walzer 2007). Walzer therefore identifies Yemen as an “in-between” zone where a “secret war” was taking place. The secrecy refers to both the ambiguity of the applicable rules and to the “secret” information about what exactly is going on in the in-between zone.

Here we see an analogy between the in-between zone and the digital sphere. Just like in the “secret war,” what exactly happens in the digital sphere and which rules apply there remain ambiguous. For example, states are unclear about how often cyberattacks happen and what exactly their effects are (Ministerie van Justitie en Veiligheid 2020). Additionally, there are no clear borders online. This has to do with the fact that a lot of citizens’ data are spread out and stored in different digital territories. Google is, for instance, a US-based company, but it also stores data of non-US citizens. If this company is hacked and citizens’ data are published, who exactly is under attack? Is it the digital territory of all states whose citizens’ data have been made public? Or is it merely the state where the company is situated? This example shows the difficulty of determining whose digital territory is under attack and therefore also the difficulty of distinguishing a separation between states’ digital zones.

As illustrated above, Walzer's concept of in-between zones aligns well with the characteristics of the digital sphere. Nonetheless, it remains an open question whether we should consider the digital sphere entirely as an in-between zone or rather see each specific country’s digital sphere to be an in-between zone.

## Conclusion

“Cyberwarfare has several unusual features. It is arguably the first major new form of warfare since the development of nuclear weapons and intercontinental missiles.” (Dipert 2016, 385)

In a world that is becoming increasingly more digital, ever more aspects of life are moving to the digital sphere, including less than pretty facets of our societies. Cyberwar is a new phenomenon; some would say unlike anything we have ever seen before. Our society is transforming and is trying to make sense of this new world of the digital. In that process, we must explore whether the theories that we have previously used are still applicable and relevant. In the case of war, for decades, Just War Theory has been a major player in defining what is just and unjust. The main question for this article was whether Just War Theory is built to meet the new phenomenon of cyberwarfare. Specifically, it looked at Michael Walzer’s theory on different zones of war, zones of peace, and in-between zones, and applied this to the digital sphere.

Upon examining the rules that govern a peace zone, it has become apparent that in most Western nations, the conditions of the peace zone are met in the physical sphere but not equally well in the digital sphere. The digital sphere is experiencing serious issues when it comes to protecting privacy, and with new laws that regulate online posting, freedom of speech is also not guaranteed. When considering whether we should view the digital sphere as a zone of war, it appears that Walzer’s theory about aggression can be applied in different ways. It seems that all types of cyberattacks, including data theft, can be seen as acts of aggression if we define aggression as an act that violates the political sovereignty or territorial integrity of a state. However, in distinguishing between zones of war and zones of peace, Walzer specifically considers “soldiers on the streets” as a crucial part of a war zone. Consequently, a digital zone of war would presumably require cyberattacks that do harm to physical property or human lives, excluding issues like data theft.

As cyberwarfare constitutes neither a zone of war nor a zone of peace, the digital sphere seems to be placed in an in-between zone. The secrecy of many such attacks, such as the one Walzer (2007) describes in Yemen, can be found also in the digital sphere, where countries are hesitant to be open about what happens online. However, Walzer is not clear about the *rules* that constitute this in-between zone. Future research should further develop rules for the in-between zone of the digital, to help us towards a clearer guide about when online aggressive behavior is just or unjust.

Furthermore, when it comes to future research, academia should pay special attention to the analogies that we use. A substantial amount of literature on cyberwar uses analogical reasoning, wherein they compare cyberattacks with attacks that happen in a conventional war (Dipert 2016). However, as this article shows, this might not always be an appropriate comparison to make. While there might be overlapping targets, goals, and indeed ethics, there are also very distinct features that play a role in cyberattacks. An example of this is the violation of the privacy of citizens in another country. Furthermore, since in conventional war the emphasis is on damage to human lives and physical objects, the comparison with the effects of cyberwar makes many types of effects of cyberattacks seem non-significant. This is not to argue that future research should no longer use the analogy to conventional war; however, it should not be restricted by it. Let us not focus only on those attacks where we can see a direct comparison with conventional war but focus more on the cyberattacks that are distinctive for cyberwar. Additionally, future research might want to explore more thoroughly whether we have a “*state* of war” or not, rather than whether we are a “zone of war.”

With all the new digital developments, are we getting closer to what we might call the “expiration date” of Just War Theory? As shown, Walzer's version of the theory – or more broadly of the tradition – is still applicable in and relevant for current affairs. However, it has its difficulties. Walzer’s conception of a war zone is too focused on the physical effects of war and therefore misses some of the important aspects of cyberwar, such as data theft. It seems, therefore, that the digital sphere must be considered an in-between zone. If we want to develop rules for an ordered and secure digital future, it is thus important to think differently about the distinction between zones of war and peace, and also to develop *jus in bello* rules for the in-between zones. I believe that the expiration date of Just War Theory is not yet here, but I believe it needs to rise to the occasion when it comes to digital warfare in order to avoid showing signs of spoil.
